# Plant based synthesised silver nanoparticles delivering enhanced antifungal activity and synergistic environmental remediation

**DOI:** 10.1038/s41598-026-40674-5

**Published:** 2026-02-23

**Authors:** Shani Raj, Rohini Trivedi

**Affiliations:** Plant Pathology Laboratory, Department of Botany, Mohanlal Sukhadia University, Udaipur, Rajasthan 313001 India

**Keywords:** Biological techniques, Biophysics, Microbiology, Plant sciences, Environmental sciences, Nanoscience and technology

## Abstract

This study delves into the antifungal capabilities and catalytic dye degradation efficiency of silver nanoparticles (AgNPs) synthesised using *Enicostemma axillare* leaf extract. Highlighting the pressing need for sustainable agriculture and pollution mitigation strategies, our investigation demonstrates the dual utility of phyto-synthesised AgNPs. Antifungal assays revealed substantial inhibition of mycelial growth and spore germination in *Alternaria alternata* and *Fusarium oxysporum*, with inhibition rates peaking at 83% and 82.79%, respectively, at a concentration of 120 µg/ml. Additionally, AgNPs showcased remarkable catalytic degradation of hazardous dyes methylene blue, methyl orange, and Congo red, achieving over 90% degradation within minutes, underlying their potential in environmental remediation. The catalytic reduction showcased not only the high efficiency of these nanoparticles in breaking down complex organic molecules but also positioned them as viable candidates for treating industrial effluents. The increased phenolic and proline contents in treated tomato plants suggest an enhanced stress response, potentially contributing to disease resistance. These findings underscore the versatility of phyto-synthesised AgNPs, offering promising avenues for the development of eco-friendly solutions in agriculture and environmental management.

## Introduction

Across the globe, the menace of phytopathogenic microorganisms to agriculture is profound, leading to a variety of diseases that significantly diminish crop yields^1^. This decline in agricultural productivity poses a looming threat to worldwide food security. A diverse array of fungi, bacteria, and viruses is known to secrete toxic substances, adversely affecting the health and output of crops^2^. While synthetic chemicals have traditionally been the go-to solution for addressing these pathogenic challenges, their broad application raises concerns over environmental safety and the well-being of non-target species. This scenario necessitates the development and adoption of novel, precise, and eco-friendly interventions to effectively counteract the threats posed by plant pathogens^3^.

The tomato, known as the ‘poor man’s apple’, is globally cultivated and widely consumed. India is the second-largest producer after China, with significant production figures. However, its cultivation faces major challenges due to biotic factors, notably Fusarium wilt and leaf blight, caused by *Fusarium oxysporum* and *Alternaria alternata*, respectively^4^. These diseases are globally significant, causing substantial yield losses up to 60–70% under conducive conditions, due to the pathogens’ ability to persist in soil and infect crops^5^. Tomato cultivation in India faces significant challenges due to its vulnerability to various diseases and parasites, notably leaf spot caused by *Alternaria alternata* and Fusarium wilt^6^. These fungal pathogens have a broad impact, affecting many crops globally and leading to substantial losses in yield and plant health, with eggplant being notably susceptible^7^. The use of methyl bromide, once the most effective control method for diseases like Verticillium wilt, is now banned in many countries due to its environmental toxicity. This prohibition has spurred interest in alternative, less harmful disease management strategies, emphasising the critical need for sustainable agricultural practices in tomato cultivation^8^.

Metal nanoparticles (MNPs), including Ag, Au, ZnO, Cu, Fe, Pd, and TiO_2_, have emerged as transformative agents in combating antibiotic resistance and advancing technologies across pharmaceuticals, electronics, photonics, sensing, therapeutics, and antimicrobial applications^9,10^. Among these, silver nanoparticles (AgNPs) have garnered significant attention due to their multifaceted properties, including antibacterial, antifungal, antiviral, antioxidant, anti-inflammatory, and anticancer activities. Their efficacy stems from mechanisms such as disrupting microbial cell membranes, generating reactive oxygen species (ROS), and interfering with DNA replication and protein synthesis, making them potent against antibiotic-resistant pathogens^11^. In addition to their biomedical potential, MNPs interact with plants, eliciting both beneficial and detrimental morphological and physiological changes influenced by their composition, concentration, size, physico-chemical properties, and the plant species involved^12^. These interactions can enhance growth or stress tolerance but may also cause toxicity. In medicinal and horticultural fields, MNPs, particularly AgNPs, are valued for their minimal toxicity, sustained activity, and high efficacy against phytopathogens, positioning them as promising tools for disease management and sustainable agricultural solutions. Silver nanoparticles (AgNPs), in particular, stand out for their exceptional antimicrobial, antioxidant, and antifungal capabilities, offering superior cytotoxic activity against a wide array of microorganisms compared to other MNPs^13^.

The synthesis of silver nanoparticles (AgNPs) involves various methods, including chemical and physical approaches, which often present challenges such as high costs, lengthy processes, and environmental hazards. To address these issues, green synthesis emerges as a viable, eco-friendly alternative, leveraging biological materials like bacteria, yeast, algae, fungi, and particularly medicinal plants for their inherent therapeutic qualities and bioactive compounds^14^. This approach not only simplifies the biosynthesis process but also aligns with the therapeutic use of medicinal plants in disease control and treatment. Specifically, *Enicostemma axillare*, a medicinal plant abundant in India and rich in flavonoids, terpenoids, and alkaloids, demonstrates significant potential for the stable synthesis of AgNPs^15^, prompting further investigation into its efficacy in green synthesis methodologies.

In contemporary times, the pervasive use of organic dyes across various industries, including textiles, paper, food, drugs, cosmetics, leather, and printing, has significantly contributed to the contamination of water bodies. These industries frequently discharge large volumes of effluents laden with organic dyes into aquatic environments without adequate treatment, posing grave environmental and health hazards^16,17^. Given their toxicity, carcinogenic properties, and resistance to degradation, these substances can cause a range of serious health issues, such as skin disorders, liver and kidney damage, and neurological poisoning^18^. The quest for effective removal of these pollutants from water has highlighted the limitations of current approaches, which are often prohibitively expensive and inefficient. This has led to a growing interest in exploring eco-friendly and cost-effective alternatives, with a particular focus on the catalytic reduction of organic dyes using biologically synthesised nanoparticles^19^. Among these, the application of silver nanoparticles has shown promise, yet research in this domain, especially concerning the treatment of dye effluents, remains in its nascent stages^20^. There is a pressing need for further studies to evaluate and refine these innovative strategies, aiming to develop sustainable solutions for mitigating water pollution caused by industrial dyes^21^.

This study examines the morphological, structural properties and antifungal effects (both *in vitro* and *in vivo*) of AgNPs synthesised using *Enicostemma axillare* leaf extract, noting its cost-effectiveness and speed. It highlights the unique application of these biosynthesised AgNPs in horticulture, specifically demonstrating their significant antifungal impact on tomato growth under controlled and natural conditions. Additionally, the study investigates the use of AgNPs as catalysts in the degradation of various organic dyes, showcasing their versatility and potential in environmental remediation.

## Materials and methodology

The experimental details and methodology are summarised in Table 1, and the following sections are outlined here.

**Table 1:** Experimental detail.

Experiment	Analysis/method	Remark
Synthesis of Ag nanoparticles	Green synthesis using the plant aqueous extract of *Enicostemma axillare*	Ag nanoparticles were successfully synthesised
Characterisation of Ag nanoparticles	XRD, TEM, SEM–EDS, DLS, FTIR	Conducted to evaluate the physicochemical properties of nanoparticles
Seedling bioassay	Blotter test method	Assessed the efficacy of nanoparticles in promoting seedling growth
Antifungal activity of Ag nanoparticles	Spore germination assay, pot experiment, poison food technique	Multiple experiments were conducted to evaluate the antifungal efficacy of Ag nanoparticles against *Alternaria* and *Fusarium* in tomato

### Materials

**Fig. 1:**
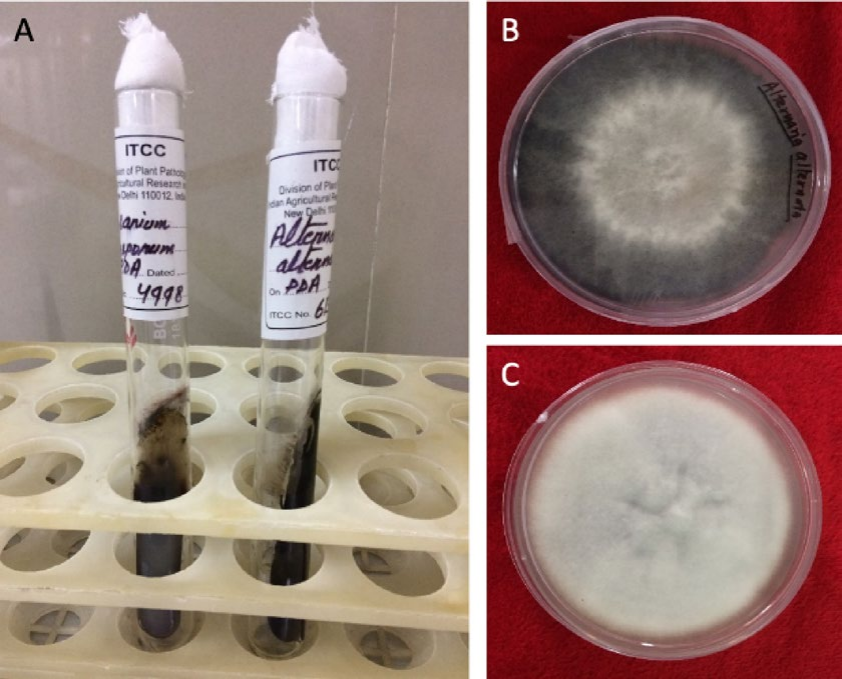
(**A**) Fungal cultures (**B**) *Alternaria alternata* and (**C**) *Fusarium oxysporum.*

AgNO_3_ (Mol. Wt. 169.87 g mol^−1^) was procured from Sigma-Aldrich, St. Louis, MO, USA. Chemicals for enzyme assay and other experiments were procured from HiMedia, CDH and SRL, Mumbai, India. The tomato seeds of cultivar ‘Raja-1’ were obtained from “Shree Udaipur Krishi Kendra”. The pure cultures of *Alternaria alternata* (ITCC No. 6134) and *Fusarium oxysporum* (ITCC No. 4998) were procured from ITCC ICAR-Indian Agricultural Research Institute, New Delhi (Fig. 1). The obtained pure fungal cultures were maintained by subculture using potato dextrose agar medium.

### Synthesis of silver nanoparticles

AgNPs were obtained as dry powder from the green synthesis method and characterised by various analytical techniques in our earlier study^22^. The obtained AgNPs were sonicated to get an equally dispersed aqueous solution.

### In vitro antifungal activity of AgNPs

Poison food technique was used to assess the antifungal activity of biosynthesised AgNPs against *Alternaria alternata* and *Fusarium oxysporum*^23^. A stock solution of 120 µg/ml was prepared by dissolving 12 mg of nanoparticles in 100 mL deionised water and was kept for sonication till a homogenous solution was obtained. From the stock solution, different concentrations such as 10, 40, 80, and 120 µg/ml aqueous solution were prepared and used in antifungal activity test against fungus species viz. *Alternaria alternata* and *Fusarium oxysporum*. Potato dextrose agar medium (HiMedia, Mumbai, India) was prepared and poured into pre-sterilised glass Petri dishes (Borosil 90 × 15 mm Culture Petri dish) separately, with the above-mentioned percentages of nanoparticles. The mycelial bit was then taken from the peripheral end of the uniform scale (diameter, 6.0 mm) from the 7-day culture of test pathogens and placed at the middle of the Petri dish test. Along with these, 100 µg/ml AgNO_3_, Mancozeb, and Bavistin solutions were also prepared and used for antifungal assay to study the comparative analysis of antifungal activity. All the Petri dishes were incubated for 10 days at 28 ± 2 °C, and radial mycelial growth was recorded when the maximum growth (90 mm) of the control Petri dish cover was recorded. All the treatments consisted of three replications, and the experiment was repeated twice. To measure the percent inhibition rate of mycelia of the pathogen using the formula given by Vincent^24^, the inoculated plates were compared with a control (without nanoparticles).

$$\% {\text{ Inhibition rate}}\, = \,\left( {{\mathrm{M}}_{{\mathrm{c}}} - {\mathrm{M}}_{{\mathrm{t}}} /{\mathrm{M}}_{{\mathrm{c}}} } \right)\, \times \,{1}00.$$

where M_c_ is the mycelial growth in control, and M_t_ is the mycelial growth in treatment. A fungal culture that showed maximum inhibition was taken for further studies.

### Spore germination method

**Fig. 2:**
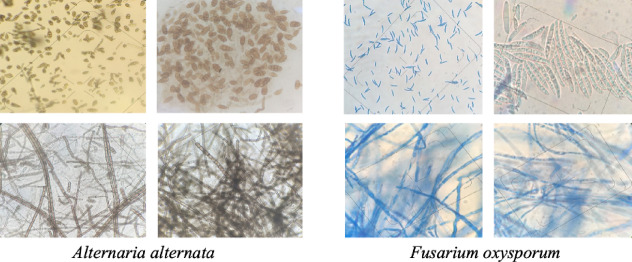
A representative microphotograph of fungal spores and mycelium of (**A**) *Alternaria alternata,* (**B**)* Fusarium oxysporum.*

The antifungal activities of nanoparticles (10, 40, 80, and 120 µg/mL) on spore germination of *A. alternata* and *F. oxysporum* were examined. Spore suspension (4.0 × 104 spores/ml) of *A. alternata* and *F. oxysporum* was prepared aseptically from a 7-day-old culture maintained on PDA (Potato Dextrose Agar) at 28 ± 2 °C. Figure 2 shows microscopic images of spores of *A. alternata* and *F. oxysporum*. The number of spores/ml was counted with the help of a haematocytometer. In 10 replicates, 50 ml of spore suspension and 50 ml of nanoparticle at the aqueous concentrations referred to above were taken on glass slides (90 × 15 mm Petri dish, Borosil). All treatments were maintained for 10 h at 28 ± 2 °C, and microscope observations were made to measure the percent inhibition rate by counting the number of spores germinated as compared to the control.

$$\% {\text{ Inhibition}}\;{\mathrm{rate}}\, = \,\left( {{\mathrm{G}}_{{\mathrm{c}}} - {\mathrm{G}}_{{\mathrm{t}}} /{\mathrm{G}}_{{\mathrm{c}}} } \right)\, \times \,{1}00.$$

where G_c_ is the germination in control and G_t_ is the germination in treatment.

### Effect of biosynthesised AgNPs on seedling growth

The tomato cultivar was used to test the effect of nanoparticles on seedling growth using standard, slightly changed methods^25^. Briefly, the tomato seeds were surface sterilised by dipping in 0.01% mercuric chloride for 30 s and immersing in 10% sodium hypochlorite solution for 10 min, and then rinsed three times with deionised water. The seeds were soaked in deionised water (control), AgNO_3_ (100 µg/ml) and biosynthesised AgNPs at different concentrations (10, 40, 80 and 120 µg/ml) for 1 h. The treated seeds were placed in Petri plates (90 × 15 mm Culture Petri dish, Borosil), containing filter paper moistened with 5.0 ml of sterilised deionised water. Each plate contained 10 seeds, and experiments were performed in triplicate for each treatment. In a dark growing room, sealed Petri plates were maintained at 28 ± 2 °C. Deionised water was applied to the Petri plates for a two-day interval to maintain the appropriate level of humidity. When seeds have shown at least 1.5–2.0 mm shoots, percent germination was noted. After 10 days of growth, percent germination, seedling length, shoot/root length, and fresh and dry weights were measured.

$$\% {\text{ Germination}}\, = \,\left( {{\mathrm{Number}}\;{\mathrm{of}}\;{\mathrm{seeds}}\;{\mathrm{germinated/Number}}\;{\mathrm{of}}\;{\mathrm{seeds}}\;{\mathrm{plated}}} \right)\, \times \,{1}00.$$

Seedling vigour index (SVI) was calculated according to the formula described by Abdul-Baki and Anderson (1973)^26^.

$${\mathrm{Seedling}}\;{\mathrm{vigor}}\;{\text{index }} = {\text{ }}\left( {{\text{germination }}\% } \right){\text{ }} \times {\text{ }}\left( {{\mathrm{seedling}}\;{\mathrm{length}}} \right)$$

## Pot experiment for the study of antioxidant and defence enzymes

### Preparation of pots and soil

The standard clay-type soil was collected from the fallow land of the research field of Maharana Pratap University of Agriculture and Technology, Udaipur, India. Pots were sterilised with 20% CuSO_4_ solution and filled with soil. To maintain the porosity and aeration in the soil, sand and cocopeat were also added to it. Pot experiments were performed in a net house under natural light and temperature conditions. Initially, seeds were sterilised by immersing them in 10% sodium hypochlorite solution for 10 min. then rinsed three times with deionised water. Seeds were germinated in the seedling tray containing cocopeat. The healthy tomato seedlings with 4–6 true leaves were transplanted to earthen pots filled with surface soil after fifteen days. After 35 days, the entire aerial part of the tomato plants was sprayed with an aqueous conidial suspension of *A. alternata* to initiate leaf spot disease. Inoculated plants were covered with a transparent plastic bag for 48 h to maintain high humidity. Foliar spray (10 ml/plant) of water (control), 100 µg/ml AgNO_3_ and different concentrations (10, 40, 80 and 120 µg/ml) of AgNPs as aqueous suspension were uniformly applied after 3–4 days of inoculation with pathogenic fungi. As a positive control in the tests, commercial fungicides (Mancozeb and Bavistin) at 100 µg/ml were also used, and a minimum of 10 plants were used in each treatment. The activities of key enzymes, including superoxide dismutase (SOD; EC 1.15.1.1), phenylalanine ammonia lyase (PAL; EC 4.3.1.5), peroxidase (POD; EC 1.11.1.7), and polyphenol oxidase (PPO; EC 1.10.3.1), were quantified in flag leaves three days after foliar application using established protocols^27–30^. Simultaneously, the leaf’s proline, total phenol, and total chlorophyll (a and b) contents were determined using standardised procedures^31–33^ during the same assessment period.

## Catalytic degradation of dyes

Catalytic potential of synthesised AgNPs was evaluated by degrading different carcinogenic organic dyes (MO, MB and CR) in the presence of NaBH_4_. Before the degradation process, the synthesised AgNPs were sonicated using ultra prob sonication for a few minutes to make an aqueous colloidal suspension. For reduction, a colloidal suspension of AgNPs and NaBH_4_ was mixed with an aqueous solution of dyes. The detail of the concentration and volume of the reaction mixture is given in Table 2. The reduction process was carried out in a 4 mL quartz cuvette, and time-dependent reduction time was observed by recording absorption spectra in a time interval of 1 min. Reaction mixture without AgNPs was used as reference, and pseudo-first-order kinetics was analysed to evaluate the rate constant as per the following equation:

1 $${\mathrm{ln}}\left( {{\mathrm{A}}_{{\mathrm{t}}} /{\mathrm{A}}_{0} } \right) \, = \, - {\mathrm{kt}}$$

**Table 2:** Effect of AgNPs on *in vitro* mycelial growth of *Alternaria alternata* and *Fusarium oxysporum*.

Sr. no	Treatment	% inhibition of mycelial growth	
		*Alternaria alternata*	*Fusarium oxysporum*
1	Control	0.00 ± 0.0^e^	0.00 ± 0.0f.
2	AgNO_3_	81.9 ± 1.7^a^	72.6 ± 1.7^c^
3	Bavistin	57.0 ± 1.7^b^	100.0 ± 0.0^c^
4	Mancozeb	67.8 ± 1.1^c^	76.3 ± 1.7^a^
5	AgNPs		
	10 µg/ml	51.9 ± 1.3^d^	49.6 ± 2.3^e^
	40 µg/ml	60.7 ± 2.8^c^	54.1 ± 1.7^e^
	80 µg/ml	72.1 ± 2.2^d^	62.5 ± 3.3^d^
	120 µg/ml	83.0 ± 0.6^a^	82.6 ± 1.3^b^

Each value is the mean of two triplicate experiments. Mean ± SE followed by the same letter in the column of each treatment are not significantly different at *p* = 0.05 as determined by IBM SPSS Statistics.

The % degradation of the dyes was estimated through the following equation

2 $${\text{Percent degradation}} = \frac{{A_{t} - A_{0} { }}}{{A_{0} }} \times 100$$

where *A*_*0*_ is the initial absorbance of dye, *A*_*t*_ is the absorbance of dye at time *t,* and *k* is the rate constant. The whole reaction of degradation was processed at room temperature.

After oxidation, the mixture was centrifuged at 10,000 rpm for 10 min, and AgNPs were separated from the mixture by filtration. 5 cycles of measurements were performed to test the reusability of AgNPs.

## Results and discussion

### Antifungal activity of synthesised nanoparticles against *Alternaria alternata*

**Fig. 3:**
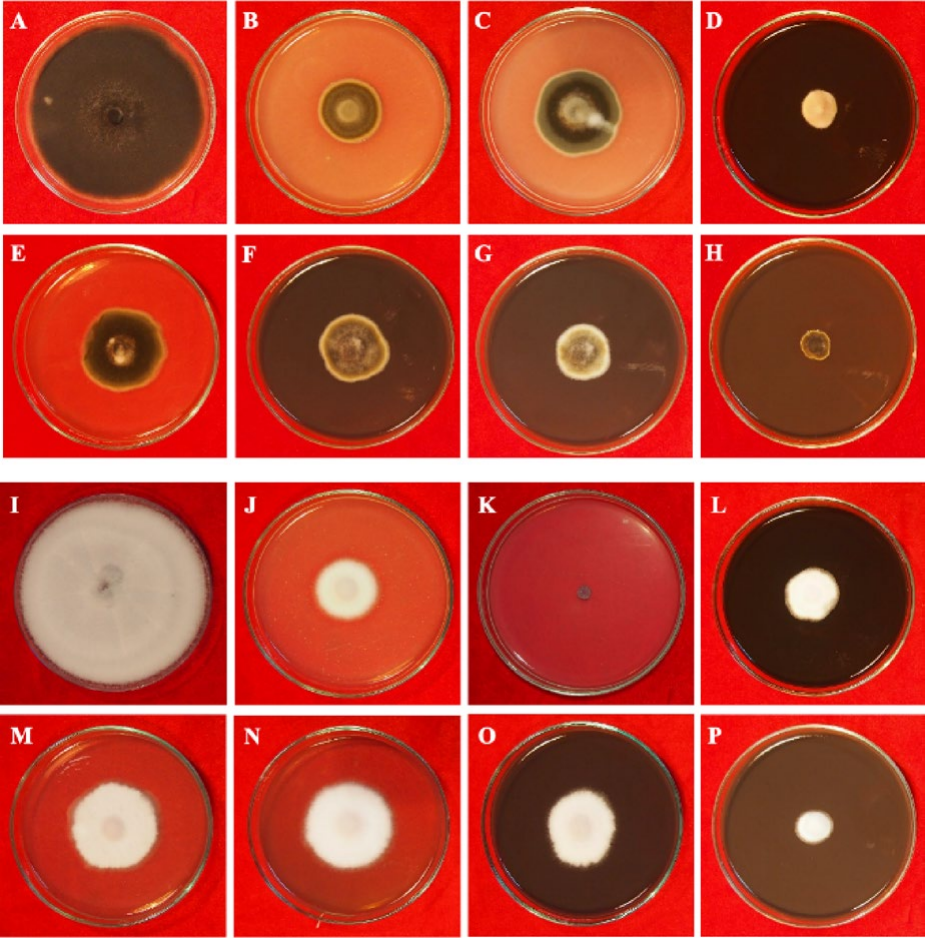
A representative photograph of an *in vitro* antifungal bioassay. Effect of AgNPs on mycelial growth of *A. alternata* (**A**) Control. (**B**) Bavistin (**C**), Mancozeb (**D**), AgNO_3_ (**E**–**H**) 10, 40, 80 and 120 μg/ml of AgNPs. Effect of AgNPs on mycelial growth of *F. oxysporum* (**I**) Control (**J**) Bavistin (**K**) Mancozeb (**L**) AgNO_3_ (M–O) 10, 40, 80 and 120 μg/ml of AgNPs.

Results of the *in vitro* antifungal activity of biosynthesised AgNPs against *A. alternata* are depicted in Table 2 and Fig. 3. AgNPs at different concentrations (10, 40, 80, and 120 µg/ml) along with bulk (AgNO_3_ 10 µg/ml), fungicides (Mancozeb and Bavistin 100 µg/ml) were used to assay the antifungal activity. At 120 µg/ml concentration of AgNPs, a maximum 83% inhibition rate was observed, followed by 80 µg/ml concentration of AgNPs (72.1%). AgNPs at lower concentrations of 10 and 40 µg/ml show non-significant antifungal activity in comparison to fungicides. The fungicides Macnozeb and Bavistin at 100 µg/ml concentration inhibit 67.8% and 57% of the fungal growth, respectively. Similarly, silver nitrate also shows high antifungal activity (81.9%) as AgNPs at 120 µg/ml, but silver nitrate is very toxic. In this study, amongst various concentrations of AgNPs, 80 µg/ml and 120 µg/ml show significant inhibition of mycelial growth of *A. alternata* as compared to fungicides.

### Antifungal activity of synthesised nanoparticles against *Fusarium oxysporum*

*In vitro* mycelial growth of *F. oxysporum* was comprehensively controlled by different concentrations of biosynthesised AgNPs shown in Table 2 and Fig. 3. In the *in vitro* antifungal assay, AgNPs at different concentrations (10, 40, 80, and 120 µg/ml) along with bulk (AgNO_3_ 100 µg/ml), fungicides (Mancozeb and Bavistin 100 µg/ml) were used. A maximum growth inhibition (82.6%) was observed at 120 µg/ml concentration of AgNPs, and minimum inhibition of 49.6% and 54.1% were recorded for 10 and 40 µg/ml AgNPs, respectively. Among the fungicides, Bavistin showed the highest percentage of mycelial growth inhibition, in which no growth was detected. Mancozeb also showed significant growth inhibition (76.3%) of *F. oxysporum* was observed at 100 µg/ml concentrations. In the case of silver nitrate (72.6%), mycelial growth was inhibited at 100 µg/ml concentration. In this study, amongst different concentrations of AgNPs, 120 µg/ml shows significant inhibition of the mycelial growth of *F. oxysporum* as compared to fungicides and bulk.

Thus, after examining the antifungal activity of *E. axillare* leaf extract-mediated synthesis of nanoparticles against *A. alternata* and *F. oxysporum*. It was observed that AgNPs showed significant activity against *A. alternata* in comparison to that of *F. oxysporum*. So, *A. alternata* was considered a test pathogen and was taken for further studies.

### Spore germination

The effect of biosynthesised AgNPs on spore germination of *A. alternata* and *F. oxysporum* is shown in Table 3. In the case of *A. alternata,* a maximum of 85.95% spore germination was inhibited by 120 µg/ml concentration of AgNPs, followed by 78.48% at 80 µg/ml of AgNPs. All the nanoparticles analysed were found to be effective in controlling spore germination of *A. alternata;* however, the concentration of AgNP at 80 and 120 µg/ml was found to be significant as compared to the bulk. At a level of 100 µg/ml, bulk AgNO_3_ was found to be less efficient in inhibiting mycelial growth and spore germination than synthesised nanoparticles. Similarly, in the case of *F. oxysporum,* AgNPs exhibited markedly higher inhibition of spore germination. The germination of *F. oxysporum* was effectively inhibited (82.79%) by AgNPs at 120 µg/ml, followed by 80 µg/ml of AgNPs (73.10%), among all treatments. The toxic properties of the AgNO_3_ show higher inhibition in comparison to low concentrations of AgNPs. Although there were no significant differences in results between *A. alternata* and *F. oxysporum* at 120 µg/ml concentration of AgNPs (Table 3).

**Table 3:** Effect of AgNPs on spore germination of *Alternaria alternata* and *Fusarium oxysporum.*

S. no	Treatment	% inhibition of spore germination	
		*Alternaria alternata*	*Fusarium oxysporum*
1	Control	0.00 ± 0.0^d^	0.00 ± 0.0^d^
2	AgNO_3_ 100 µg/ml	63.54 ± 3.7^b^	71.11 ± 3.8^b^
3	AgNPs		
	10 µg/ml	48.43 ± 3.4^c^	52.73 ± 3.4^c^
	40 µg/ml	60.04 ± 6.8^b^	65.86 ± 1.8^b^
	80 µg/ml	78.48 ± 3.8^a^	73.10 ± 3.1^b^
	120 µg/ml	85.95 ± 4.4^a^	82.79 ± 2.3^a^

Each value is the mean of two triplicate experiments. Mean ± SE followed by the same letter in the column of each treatment are not significantly different at *p* = 0.05 as determined by IBM SPSS Statistics.

### Seedling bioassay

**Fig. 4:**
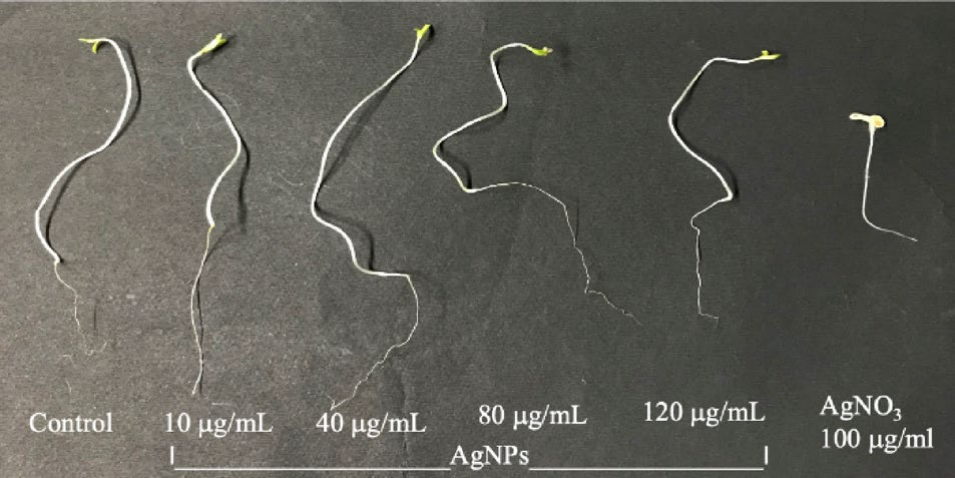
Effect of biosynthesised AgNPs on seed germination and seedling growth of tomato.

**Fig. 5:**
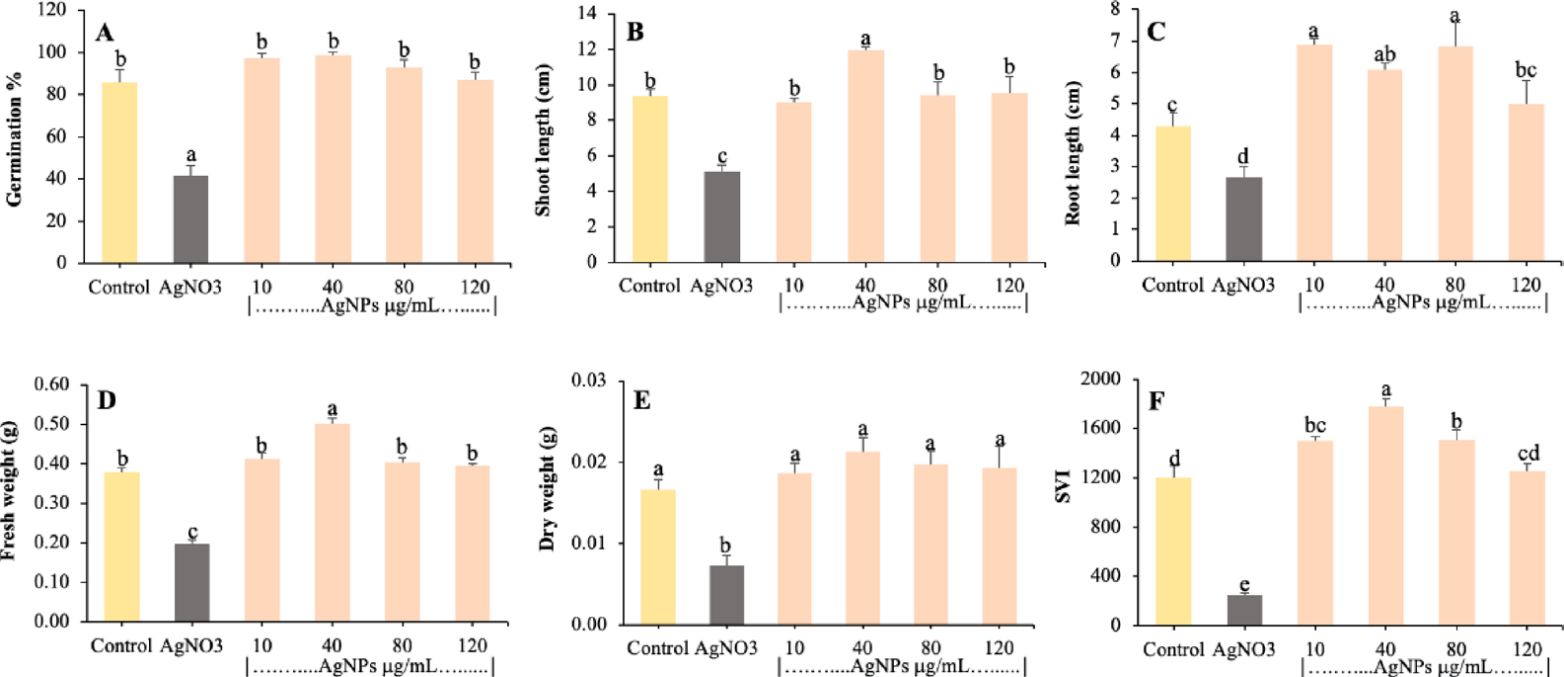
Effect of biosynthesised AgNPs on (**A**) percent germination; (**B**) shoot and (**C**) root length; (**D**) fresh weight; (**E**) dry weight; and (**F**) seed vigour index of maize seedling. Data were recorded after 10 days. Each value is the mean of triplicates, and each experiment consisted of 10 seedlings. The same letter in the graph of each treatment is not significantly different at *p* = 0.05 as determined by Tukey’s HSD, control with water.

**Fig. 6:**
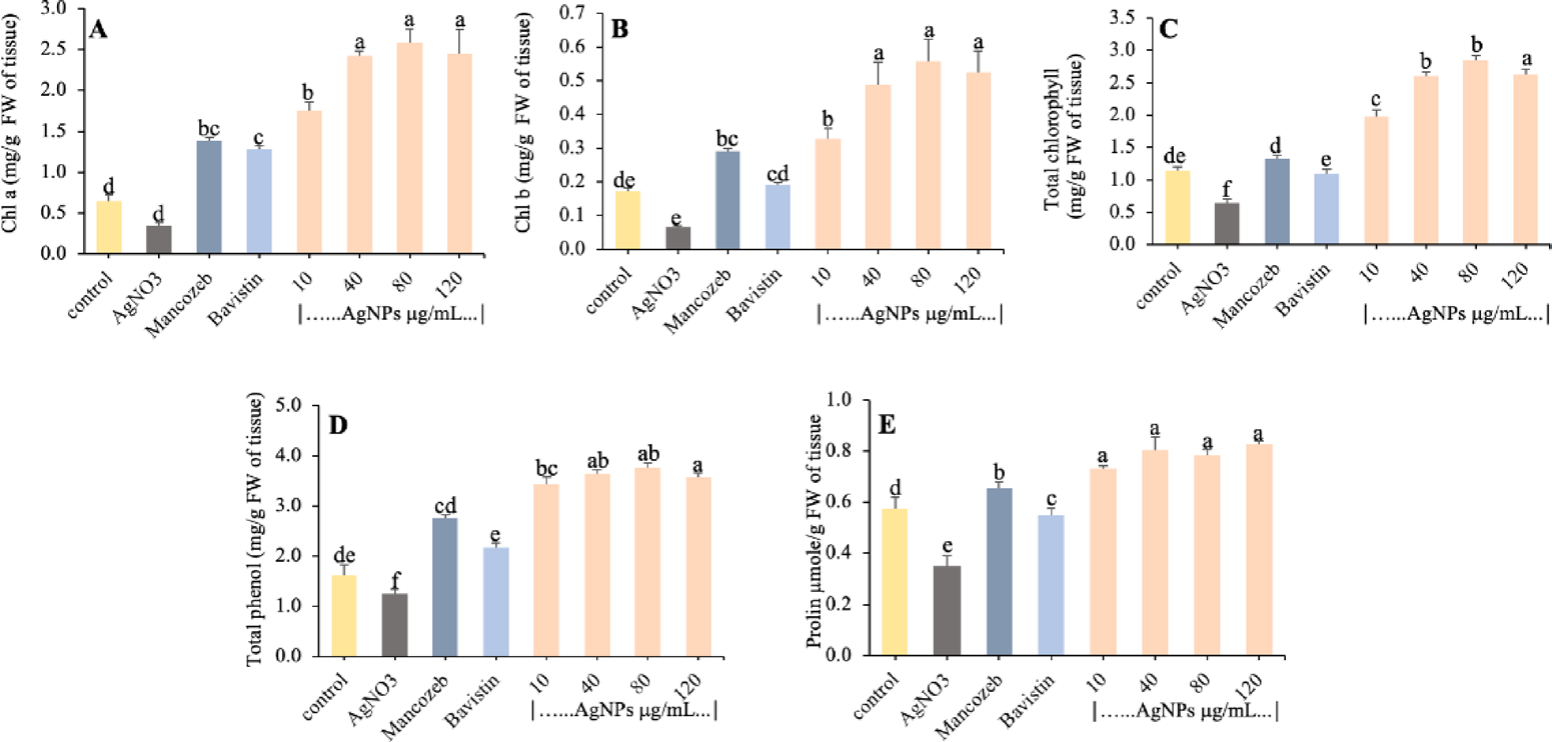
Effect of biosynthesised AgNPs on (**A**) chlorophyll-a and (**B**) chlorophyll-b, (**C**) Total chlorophyll content, (**D**) Total phenol, (**E**) Proline content. Each value is the mean of triplicates, and each replicate consisted of 3 plant samples, and the same letter in the graph of each treatment is not significantly different at *p* = 0.05 as determined by Tukey’s HSD, control with water.

Seeds treated for 1 h were grown for 10 days to study the effect of different concentrations of AgNPs on seedling growth, and data for percent germination, seedling length, root/shoot length, fresh and dry weight, and seedling vigour index (SVI) were obtained (Figs. 4, 5). The Results have shown that AgNPs vary significantly in all parameters used to calculate seedling growth. In this study, seeds treated with 10, 40, 80 and 120 µg/ml of AgNPs concentrations show significantly higher values of germination, seedling length, fresh and dry weight, and seedling vigour index as compared to all treatments. Bulk (AgNO_3_) at 100 µg/ml shows significantly lower values of germination and length of seedling. However, AgNPs at 120 µg/ml exhibit a slight decrease in germination, seedling length, fresh and dry weight, and SVI compared to the lower concentration of AgNPs but significantly higher than the control and bulk AgNO_3_ (Fig. 5). From this study, it can be concluded that all the treatments of the nanoparticles showed better seedling length in comparison to that of the control (Fig. 6). Results of the study clearly show the nontoxic effect of AgNPs on tomato seedlings, and based on these findings, we further selected these treatments to check antioxidant activities. Comparative overview of nanoparticles in antifungal activity, with some previous studies of various nanoparticles shown in Table 4.

**Table 4:** Comparative overview of nanoparticles in antifungal applications.

NPs	Source	Synthesis technique	Key findings	Antifungal application	Reference
CuNPs	*Eucalyptus* and* Mint leaves*	Aqueous extract (Green synthesis)	*Eucalyptus* leaf extract CuNP, ranging from 10 to 130 nm, and Mint leaf extract synthesised CuNP, ranging from 23 to 39 nm. At 500 ppm of CuNPs, the fungal growth inhibition was found	*Colletotrichum capsici*	^67^
ZnONPs	*Eucalyptus globulus leaves*	Aqueous extract (Green synthesis)	NPs with varied shapes and sizes ranging between 52–70 nm At 100 ppm of ZnNPs, the fungal growth inhibition was found	*Alternaria mali, Botryosphaeria dothidea,* and *Diplodia seriata*	^68^
TiO_2_NPs	*Trianthema portulacastrum, Chenopodium quinoa leaves*	Aqueous extract (Green synthesis)	NPs with spherical shapes and sizes ranging between 10–20 nm At 150 ppm of TiO_2_NPs, the fungal growth inhibition was found	*Colletotrichum graminicola*	^69^
AgNPs	*Enicostemma axillare*	Aqueous extract (Green synthesis)	NPs with spherical shapes and sizes ranging between 15–20 nm At 120 ppm of AgNPs, the fungal growth inhibition was found	*Alternaria alternata* and* Fusarium oxysporum*	Current study

### Determination of ROS scavenging enzyme assay in the pot experiment

**Fig. 7:**
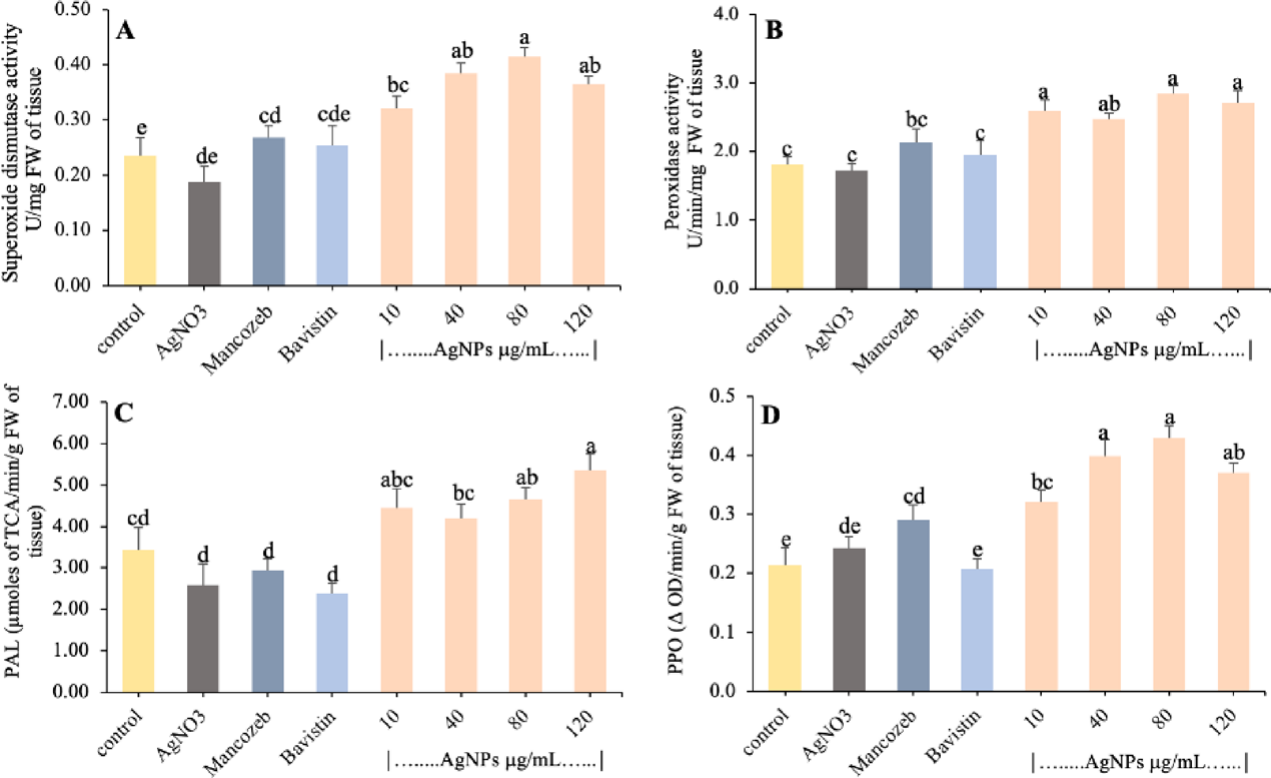
Effect of biosynthesized AgNPs on (**A**) SOD (**B**) POD (**C**) PAL (**D**) PPO enzymes activity in tomato plant leaves after three days of foliar application. Each value is the mean of triplicate, and each replicate consisted of 3 plant samples. The same letter in the graph of each treatment is not significantly different at *p* = 0.05 as determined by Tukey’s HSD, control with water.

Figure 7A shows the change in SOD activity in plant leaves exposed to various concentrations of biosynthesized AgNPs along with fungicides and bulk AgNO3 as compared to control. The amount of SOD activity was increased for all concentrations of AgNPs. Among them, the higher level of SOD at 64.2% and 77.0% were recorded at 40 and 80 µg/mL concentration of AgNPs in comparison to control and 2.02- and 2.18-fold increased than bulk AgNO_3_ respectively. The lowest activity was observed for AgNO_3_ (Fig. 7A). The activity for fungicides (Mancozeb and Bavistin) was increased by 14.4% and 8.4% than the control, but was lower than all concentrations of AgNPs.

The highest GPOD activity in the leaves was exhibited at a higher concentration of biosynthesised AgNPs. The increased activity was observed at 80 and 120 µg/ml, with an increase of 57.1% and 49.7% as compared to the control and 66.3% and 58.5% as compared to bulk AgNO_3_. The fungicides (Mancozeb and Bavistin) also show higher activity at 17.9% and 7.7% as compared to the control, and 24.8% and 14.0% higher than bulk AgNO_3,_ respectively, but it was lower as compared to each treatment of AgNPs (Fig. 7B).

In this research, quantitative changes in PAL activity in plant leaves by foliar application of different concentrations of AgNPs for plant protection were also observed. Figure 7C indicates the rate of PAL activity of the control, AgNO_3_, and each treatment of AgNPs. Maximum activity of 35.6% and 55.6% was observed in plants treated with a higher concentration of AgNPs at 80 and 120 µg/ml, respectively, in comparison to the control plant. Similarly, an increase of 80.1% and 106.7% in AgNO_3_ treatment, whereas the lowest activity of PAL was detected in fungicides (Mancozeb and Bavistin) at − 14.0% and -30.5% and bulk AgNO_3_ at -24.7% compared to the control one. For PAL activity, a wave-like pattern was observed in terms of rising and declining trends (Fig. 7C).

Excessive quantities were found in the case of PPO activity in tomatoes treated with different concentrations of biosynthesised AgNPs (Fig. 7D). At different AgNP concentrations, 50.5–73.4% activity in leaves was observed at 10–120 µg/ml compared to the control and 32–52% in comparison to bulk AgNO_3_, while maximum PPO activity was observed at 80 µg/ml, as there was an increase of 100.6% compared to control. Lower concentrations, i.e., 10–40 µg/ml, also showed higher PPO activity in comparison to the control and AgNO_3_. These findings showed that, as opposed to plants treated with fungicides, AgNPs could induce a higher amount of PPO activity.

**Fig. 8:**
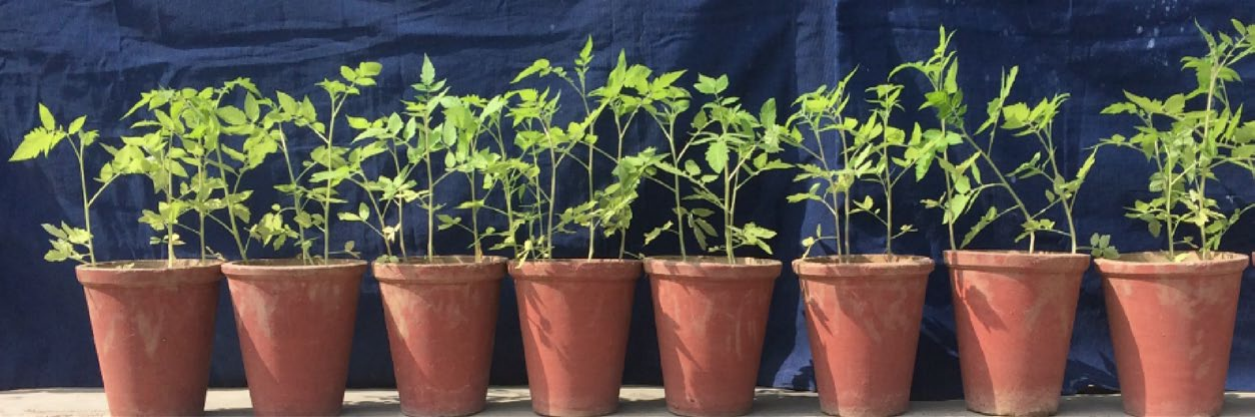
AgNPs treated tomato plants in an in vivo pot experiment.

In the present study, in the pot experiment (Fig. 8), foliar application of biosynthesised AgNPs significantly induced antioxidant enzyme activity in tomato leaves. The increased level of SOD efficiently converts toxic superoxide radicles into less toxic H2O2 species^34^, while PPO plays a key function in developing defensive resistance to plant diseases by catalyzing phenolic oxidation^35^. In AgNPs treated plants SOD and POD activity were increased which might be responsible for balancing, scavenging, and degeneration of reactive oxygen species to plants during pathogen invasion from oxidative stress. Like current findings, Kumari et al. (2019) observed that biosynthesised AgNPs can decrease the pathogenic population of *Alternaria solani*, the concentration-dependent causative agent of early tomato blight, and reported that pre-treatment of particles on leaves improved host resistance and prevented infection by raising the content of antioxidants and chlorophyll^36^. A significant rise in PPO and PAL activity at higher concentrations of AgNPs is commonly considered a measure of tolerance in tomato plants. These findings, therefore, indicate that biosynthesised AgNPs increase the amount of antioxidant enzymes that may serve as a protective mechanism against oxidative stress due to disease, whereas antioxidant defences have been correlated with healthier plant development. The plant defence system was enhanced by the improved activity of PAL, PPO, and POD enzymes^37–40^. The enhanced activity of PPO and PAL can be related to the strengthening and development of certain bioactive compounds such as lignin, suberin, quinones, and melanin that serve as a defensive shield for approaching pathogens by killing their pectolytic enzymes^41–43^. It is also proposed in the study of Haghighi Pak et al. (2017) that silver collected in root tissues after treatment with AgNP primarily occurs in the form of NPs that were extremely stable, and after splashing the cells did not release ionic silver, so they displayed less toxicity relative to ionic silver^44^.

Bio-fabricated silver nanoparticles (AgNPs) exhibit large, clear zones of inhibition against fungal plant pathogens, such as *Alternaria solani* and *Fusarium oxysporum*, due to their interaction with fungal cell walls. Phytochemicals, such as flavonoids and tannins, present as stabilising agents on AgNP surfaces, enhance antifungal activity by binding to fungal cell wall proteins, causing membrane rupture^45^. Flavonoids, known for their antifungal properties against *Candida* species and other pathogens, form complexes with extracellular proteins, weakening the fungal cell wall and halting growth^46^. Additionally, AgNPs generate reactive oxygen species (ROS), leading to oxidative stress, lipid peroxidation, and fungal cell apoptosis, consistent with prior studies^47^. Although high-temperature synthesis may degrade some phytochemicals, AgNPs’ potent antifungal effects likely stem from both ROS production and Ag⁺ ion-mediated damage to membrane proteins and DNA. These properties make AgNPs promising for bioactive agricultural applications, such as crop protection, outperforming CuNPs, ZnO NPs, and TiO2 NPs^48^. Further research is needed to elucidate the precise interactions between phytochemicals and fungal cell wall proteins to fully understand AgNPs’ antifungal mechanisms.

### Non-enzymatic parameters assessment

#### Chlorophyll content

Chlorophyll content plays an important role in the growth of plants. To estimate the photosynthetic potential of the plant, the amount of Chl a and Chl b and total chlorophyll was measured. Figure 6A–C shows that the Chl a, Chl b, and total chlorophyll content significantly increased with increasing concentration of biosynthesised AgNPs. In our result, Chl a 2.58 ± 4.04 and 2.46 ± 0.28 mg/g and Chl b 0.56 ± 0.06 and 0.53 ± 0.06 mg/g increased at 80 and 120 µg/ml concentration of AgNPs, respectively. Total chlorophyll, i.e., 2.84 ± 0.08 and 2.63 ± 0.09 mg/g, increased significantly in tomato leaves at 80 and 120 µg/ml AgNPs concentration, respectively, in comparison to the control (Fig. 6). In AgNO_3_ treatment, chlorophyll a and b and total chlorophyll content were found to be minimum (0.35, 0.07, and 0.06 mg/g). Similar results were obtained in the study of Pandey et al. (2014), who observed that the chlorophyll content was increased with increasing concentration of synthesised AgNPs^49^. In tomato plants for biosynthesised AgNPs, Farghaly and Nafady (2015) noted significant stimulation for Chl b and carotenoids, although they noted a stress effect after 35 days of treatment by measuring decreased Chl a^50^. Elbeshehy et al. (2014) studied that systemic resistance to bean yellow mosaic virus (BYMV) was induced in *Vicia faba* by the use of biosynthesised AgNPs and resulted in increased photosynthetic pigment concentration, whereas it was also reduced in infected plants^51^. The study of Ocsoy et al. (2013) also supports the role of AgNPs in the amelioration of bacterial spot disease in tomato plants (Ocsoy et al. 2013).

#### Total phenols

The key to plant immune response is phenolic compounds, which are involved in activating plant defence genes, phytoanticipin and phytoalexin production, structural barriers, and pathogenicity regulation. In the present study, total phenolic compounds were quantified at various concentrations of AgNPs. The result showed that no statistically significant difference in total phenolic content in tomato plants at different concentrations of AgNPs (Fig. 6D). The 40 and 80 µg/ml concentration of AgNPs showed a 2.26- and 2.33-fold increase in total phenolic content as compared to the control and 2.91- and 3.0-fold increase in total phenolics, respectively, in comparison to AgNO_3_ treatment. However, the activity also increased to 1.71 and 1.34-fold in fungicide treatments of Mancozeb and Bavistin, respectively. Krishnaraj et al. (2012) showed the high level of phenolics in the AgNPs-treated plants and hypothesised that plants could benefit from moderate stress in protecting them from pathogen attacks^53^. In the study of Elbeshehy et al. (2014), higher accumulation of phenolic content was observed in virus-infected bean leaves treated with AgNPs in comparison to the leaves treated with other treatments. It was obvious from our experiment that enhanced phenolic activity reduced the severity of the disease in tomato plants treated with various AgNPs concentrations, resulting in leaves of treated tomato plants being more resistant than those of control plants. Virus-infected broad bean leaves treated with AgNPs indicated maximum accumulation of phenolic contents in comparison to the infected leaves at different levels of treatments^54^. From our analysis, it was clear that the severity of the disease was decreased with increased phenolic activity in tomato plants treated with different AgNPs concentrations, so that the infected leaves of treated tomato plants were found to be more resistant than those of the control plants.

#### Proline content

Figure 6E demonstrates the effects of proline estimation in AgNPs-treated tomato plants in response to fungal infection. With increasing AgNPs concentrations, proline content was significantly increased shown in Fig. 6E. There is no statistically significant difference in proline content in AgNPs-treated plants. A maximum value was recorded at 40 and 80 µg/ml AgNPs, which were 1.38- and 1.35-fold higher than control plants and 2.36- and 2.3-fold over AgNO_3_ treatment. In this way, the proline content was increased might be due to the production of reactive oxygen species (ROS) in response to AgNPs stress. In addition, during the stress condition, the concentration of proline is enhanced and acts as a signalling molecule to modulate mitochondrial functions, cause cell proliferation or cell death and induce specific gene expression^55^. Similar findings were obtained when different concentration of copper nanoparticles was applied to the *Adiantum lunulatum* and *Arabidopsis thaliana*, the proline content was increased^56,57^. While proline content enhancement has been defined in many studies in response to NPS, the biochemical importance of this accumulation of molecules under NP stress is not well known ^58^. However, few studies have shown that this proline increase may be important for the protective mechanism against oxidative stress triggered by potentially toxic elements^59,60^. In our study, the increase in proline in tomato leaves treated with biosynthesised AgNPs could be related to the antioxidant properties of this amino acid, particularly in terms of reducing membrane and protein damage.

#### Catalytic degradation of dyes using AgNPs

**Fig. 9:**
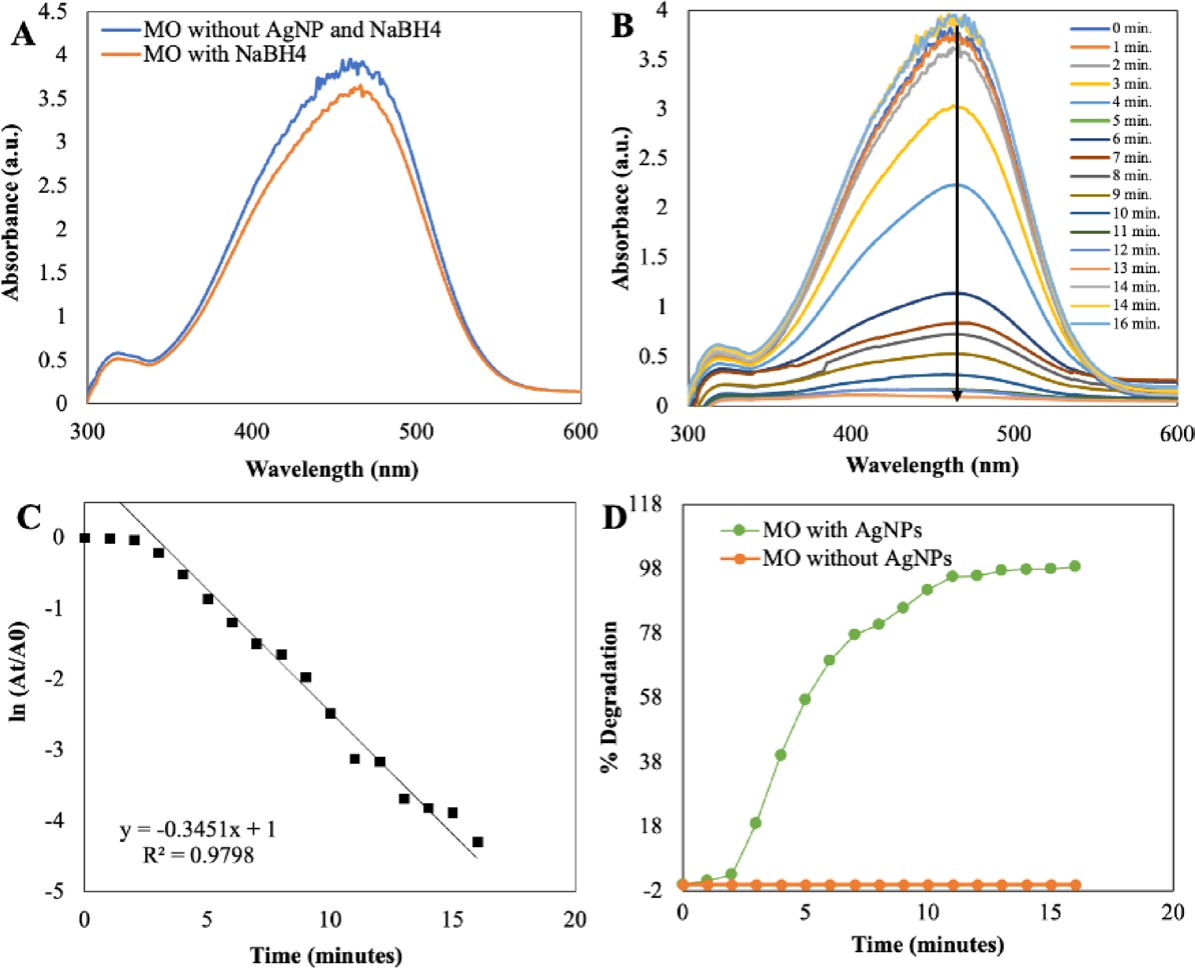
UV–visible absorption spectra analysis of dye degradation (**A**) MO without NaBH_4_ and AgNPs and in the presence of NaBH_4_ only and (**B**) Catalytic degradation of MO by NaBH_4_ in the presence of AgNPs, (**C**) Pseudo-first-order plot of ln(A_t_/A_0_) versus time of MO and (D) Percent degradation of MO over time by AgNPs.

In this study, a catalytic reduction process employing silver nanoparticles (AgNPs) in the presence of sodium borohydride (NaBH_4_) as the reducing agent was investigated for Methylene Blue (MB), Methyl Orange (MO), and Congo Red (CR) dyes. Notably, MO, a hazardous azo dye, displayed a slow degradation rate with NaBH_4_ alone (Fig. 9A). However, the introduction of AgNPs, known for their high surface area and catalytic properties, remarkably accelerated the degradation process. Initially, the MO solution exhibited an orange colour, which deepened upon NaBH_4_ addition and featured a strong UV spectral band at 464 nm, with no absorbance change upon NaBH_4_ addition. However, when AgNPs were introduced into the MO solution with NaBH_4_, a rapid reduction in absorbance was observed, leading to complete MO degradation within just 16 min (Fig. 9B). The degradation kinetics followed pseudo-first-order reaction kinetics with a rate constant (k) of 0.979 min^−1^ (Fig. 9C), underscoring the efficiency of AgNPs in catalysing MO degradation, with promising implications for environmental applications such as wastewater treatment.

**Fig. 10:**
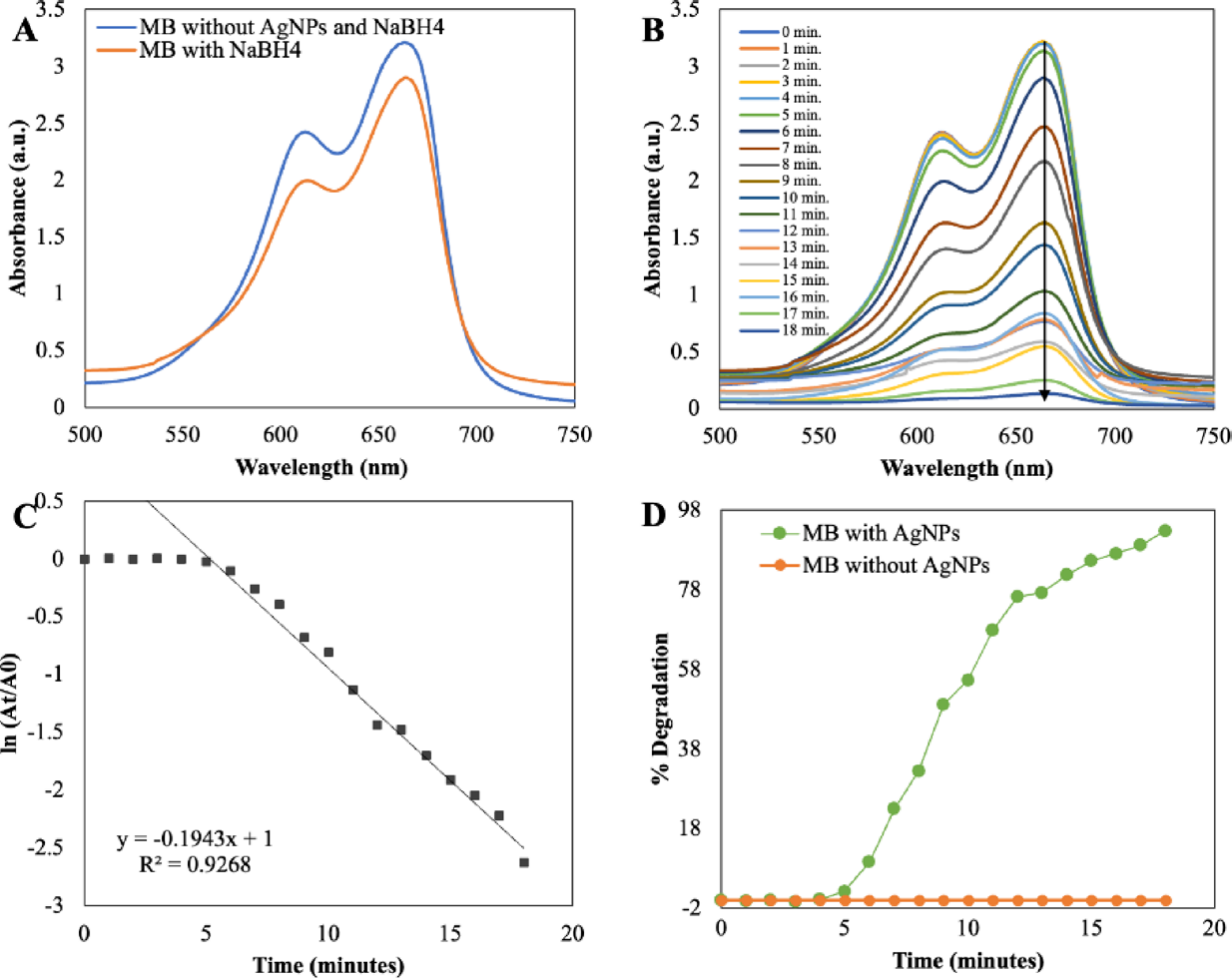
UV–visible absorption spectra analysis of dye degradation (**A**) MB without NaBH_4_ and AgNPs and in the presence of NaBH_4_ only, (**B**) Catalytic degradation of MB by NaBH_4_ in the presence of AgNPs, (**C**) Pseudo-first-order plot of ln(A_t_/A_0_) versus time of MB and (**D**) Percent degradation of MB over time by AgNPs.

Utilising silver nanoparticles (AgNPs) derived from *E. axillare*, effective degradation of Methylene Blue (MB) was accomplished. MB, a cationic heterocyclic aromatic dye, serves various purposes in analytic chemistry as a redox indicator and finds applications in the aquaculture industry as a chemotherapeutic and anti-malarial agent. Aqueous MB solutions exhibit a vivid blue colour and feature a prominent UV absorption peak at 664 nm, with an additional peak at 613 nm attributed to n → π* and π → π* transitions (Fig. 10). Intriguingly, when treated with the potent reducing agent NaBH_4_ in isolation, no degradation of MB was observed. However, the introduction of AgNPs facilitated the reduction of MB to its colourless form, leucomethyleneblue, and a consequent decline in absorbance, which was meticulously monitored spectrophotometrically at 664 nm, as depicted in Fig. 10B. This reduction process reached completion within a relatively brief span of 18 min and adhered to pseudo-first-order kinetics, with a calculated rate constant (k) of 0.926 min^−1^, as illustrated in Fig. 10C. These findings underscore the efficacy of AgNPs in expediting the degradation of MB in the presence of NaBH_4_, offering valuable insights for applications ranging from analytical chemistry to aquaculture.

**Fig. 11:**
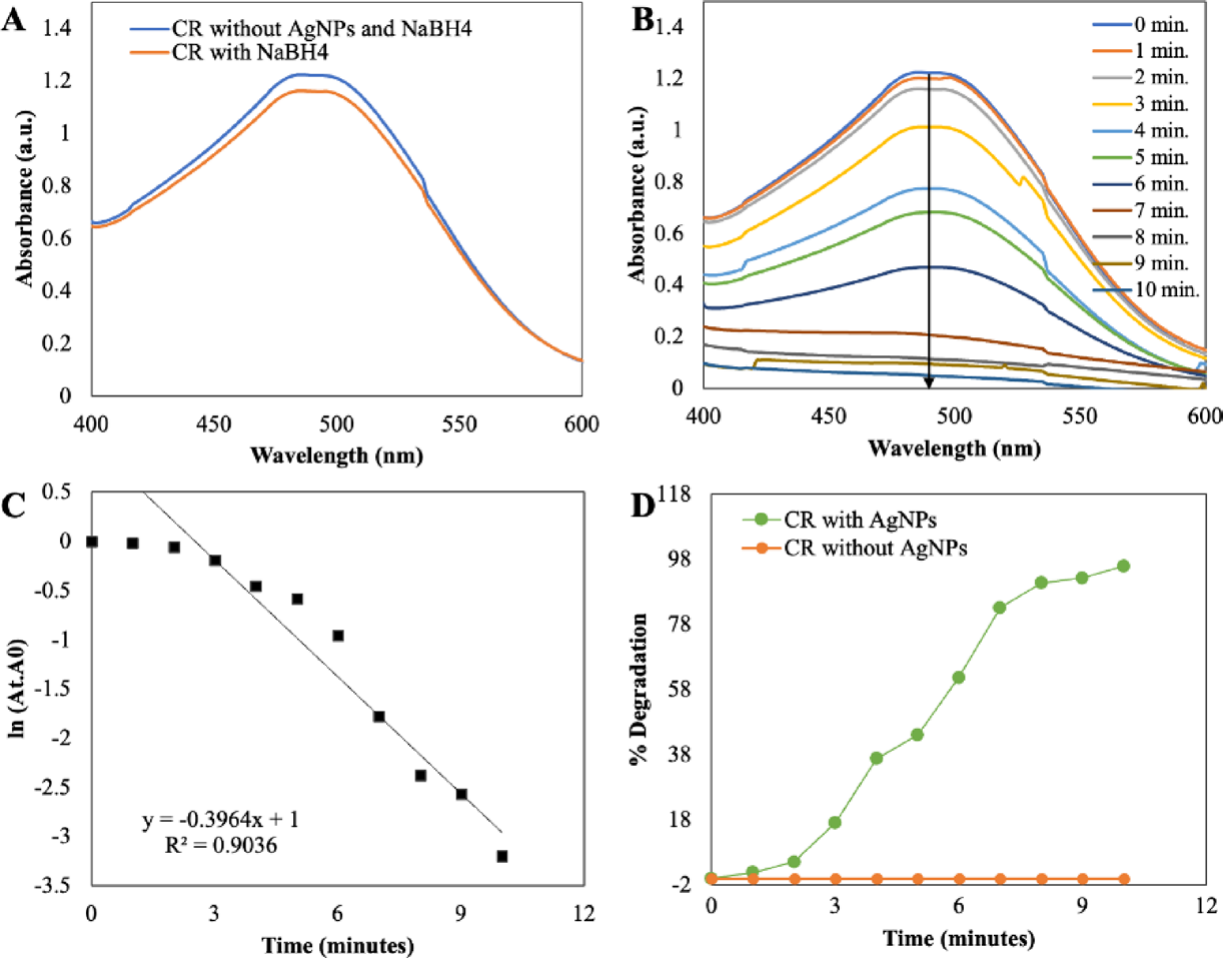
UV–visible absorption spectra analysis of dye degradation (**A**) CR without NaBH_4_ and AgNPs and in the presence of NaBH_4_ only, (**B**) Catalytic degradation of CR by NaBH_4_ in the presence of AgNPs, (**C**) Pseudo-first-order plot of ln(A_t_/A_0_) versuss time of CR and (**D**) Percent degradation of CR over time by AgNPs.

In the aqueous solution of Congo Red (CR), characterised by its vibrant brick-red colour, a prominent absorption peak was noted at 490 nm, as depicted in Fig. 11A. Intriguingly, the intensity of this absorption remained steadfast, with no discernible decolouration observed upon the addition of sodium borohydride (NaBH_4_) over an extended duration. However, the introduction of silver nanoparticles (AgNPs) into the CR solution, already containing NaBH_4_, elicited an instantaneous and pronounced reduction in absorption, as vividly illustrated in the UV spectra presented in Fig. 11B. Astonishingly, complete degradation of CR was achieved within a mere 10 min. To delve into the kinetics of this catalytic reaction, the pseudo-first-order rate kinetic law was applied, with the plot ln(A_t_/A_0_) versus reaction time pertaining to CR^61,62^ (as delineated in Fig. 11C). Consequently, the rate constant for CR degradation was determined to be an impressive 0.903 min^−1^. Furthermore, employing equation (ii) enabled the calculation of the percent degradation of the dye, revealing a staggering level of more than 90% degradation. As showcased in Fig. 9D,10D and 11D, the percent degradation for MO stood at 98.63%, while MB and CR demonstrated rates of 92.76% and 95.90%, respectively. This remarkable catalytic prowess can be attributed to the high volume-to-surface ratio inherent to the synthesised AgNPs, which furnishes an abundance of catalytic sites and lowers the activation energy. Consequently, the catalytic degradation of organic dyes likely stems from surface interactions between reactants and AgNPs, aligning with the principles of the Langmuir–Hinshelwood model^63^.

**Fig. 12:**
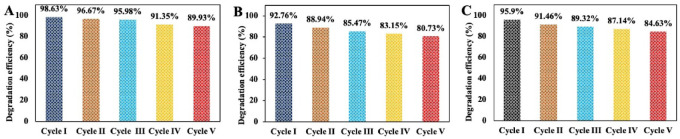
Recycling experiments of green synthesised AgNPs for catalytic degradation of dyes (**A**) MO, (**B**) MB, (**C**) CR.

AgNPs, as heterogeneous catalysts, demonstrated robust reusability across five cycles without significant loss of catalytic activity (Fig. 12). The catalytic degradation of dyes relies on the generation of hydroxyl radicals (OH·). H₂O₂ adsorbed onto AgNP surfaces accepts electrons, forming OH· radicals via OH⁻ ion oxidation. These radicals, bound to the nanoparticle surface, react with dyes, oxidising them into CO₂ and H₂O. The rate of catalytic oxidation depends on H₂O₂ adsorption and electron transfer from AgNPs, which effectively weakens the O–O bond in H₂O₂, enhancing adsorption and electron transport. Literature reports suggest that methyl orange (MO) degradation involves hydroxyl (OH·) and hydroperoxyl (HOO·) radicals derived from H₂O₂, supporting the observed mechanism^64^. This catalytic efficiency complements AgNPs’ antifungal activity, highlighting their versatility in agricultural and environmental applications.

In the context of reducing various organic dyes, the utilisation of sodium borohydride (NaBH_4_) as a reducing agent is favourable in terms of kinetics but faces thermodynamic challenges. Consequently, a range of nanocatalysts has been explored to expedite the reduction of organic dyes while ensuring kinetic feasibility. Within the mechanism underlying the catalytic reduction of these dyes, NaBH_4_ assumes a critical role by dissociating to produce BH_4_^−1^ ions, which act as electron donors, while the dye molecules function as electron acceptors. Simultaneously, silver nanoparticles (AgNPs) serve as intermediaries, facilitating the transfer of electrons from BH_4_^−1^ ions to the dye molecules. In an aqueous medium, both BH_4_^−1^ ions and dye molecules migrate toward the surface of AgNPs, where they become attached. The hydrogen released from BH_4_^−1^ ions serves as a hydrogen source, binding to the catalytic surface of AgNPs and initiating an attack on the dye molecules. The electron-carrying properties of AgNPs activate their catalytic surface, leading to the activation of azo bonds within the dyes. This activation results in the cleavage of azo bonds due to the uptake of electrons via the catalyst and the presence of hydrogen from BH_4_^−1^ ions, ultimately resulting in the reduction of the dye molecules on the surface of the nanoparticles^65,66^.

## Conclusion

This study highlights the significant potential of phyto-synthesised silver nanoparticles (AgNPs) from *Enicostemma axillare* leaf extract as an eco-friendly solution for sustainable agriculture and environmental remediation. Demonstrating substantial antifungal efficacy against pathogens like *Alternaria alternata* and *Fusarium oxysporum*, these AgNPs emerge as a promising alternative to chemical fungicides, enhancing crop resilience and yield with environmental conservation in mind. Additionally, their remarkable efficiency in catalysing the degradation of toxic dyes such as Methylene Blue, Methyl Orange, and Congo Red positions them as an effective means for mitigating water pollution. The dual functionality in disease management and pollution control underscores the role of green nanotechnology in agricultural and environmental strategies, opening new research and development avenues for eco-friendly nanoparticles. This research not only advances the scientific understanding of phyto-synthesised AgNPs but also highlights their practical applications in promoting sustainable agricultural and environmental practices.

### Statistical analysis

Data were analysed using IBM SPSS Statistics (Version 26.0, IBM Corp., Armonk, NY). Significant differences among treatment groups were determined using one-way analysis of variance (ANOVA) followed by Tukey’s HSD test at a significance level of *p* < 0.05. All experiments were conducted in triplicate, with each replicate comprising at least three samples for pot experiments and ten samples for field experiments, randomly selected from plants to ensure representative sampling.

## Data Availability

The datasets used and/or analysed during the current study are available from the corresponding author on reasonable request.
